# Antiseptic susceptibility profiles of canine pyoderma-associated staphylococci in Japan: first identification of plasmid-borne *smr* in *Staphylococcus coagulans*

**DOI:** 10.3389/fvets.2025.1621915

**Published:** 2025-09-08

**Authors:** Manami Tsunoi, Manabu Takiguchi, Emi Ashida, Kazuki Harada, Keita Iyori, Koki Shimizu

**Affiliations:** 1 Core Research Facilities, Research Center for Medical Sciences, The Jikei University School of Medicine, Tokyo, Japan; 2 Laboratory Service for Animals, 1sec Co., Ltd, Fujisawa, Kanagawa, Japan; 3 Joint Graduate School of Veterinary Sciences, Tottori University, Tottori, Japan; 4 Department of Applied Mathematics, Tokyo University of Science, Tokyo, Japan

**Keywords:** *Staphylococcus coagulans*, canine pyoderma, antiseptic reduced susceptibility, *smr*, rolling-circle replication plasmid, chlorhexidine

## Abstract

**Introduction:**

International guidelines recommend the use of antiseptics, such as chlorhexidine, to treat canine pyoderma. However, data on the antiseptic susceptibility of its primary causative agents, *Staphylococcus pseudintermedius* and *S. coagulans*, in Japan are limited.

**Methods:**

We performed antiseptic susceptibility testing and polymerase chain reaction (PCR) screening for antiseptic resistance-associated genes in these species. In addition, hybrid genome sequencing was conducted for a resistant isolate to investigate the genetic context of resistance genes.

**Results:**

Most isolates exhibited low minimum inhibitory concentrations for the tested antiseptics, although some inter-strain variations were observed. One *S. coagulans* isolate (SC18) was identified as smr-positive, representing only the second global report and the first from Japan. Phylogenetic analysis using publicly available genome data revealed that SC18 belongs to the major lineage of *S. coagulans*. Hybrid genome sequencing further demonstrated, for the first time, that *smr* in *S. coagulans* is plasmid-borne. Notably, this plasmid was also identified in a human-derived *S. epidermidis* strain (KSE124-2) in Japan, suggesting plasmid-mediated interspecies transmission between humans and companion animals.

**Discussion:**

These findings highlight the need for continued surveillance of antiseptic resistance-associated genes, which may contribute to reduced phenotypic susceptibility and pose a potential public health concern.

## 1 Introduction

Antimicrobial resistance in companion animals poses a serious health concern (1). Bacterial skin infections are among the most common reasons for systemic antimicrobial use in dogs and cats. However, emergence of methicillin-resistant *Staphylococcus pseudintermedius* and *S. coagulans* strains has raised clinical challenges (2). To address this, the International Society for Companion Animal Infectious Diseases recommends the use of antiseptics, such as chlorhexidine, as alternatives to systemic antimicrobials for canine superficial pyoderma treatment (3, 4). However, reports over the past 10 to 15 years have suggested the acquisition of antiseptic resistance-associated genes, notably *qac*, by staphylococci (5–7), highlighting the need for continuous antiseptic susceptibility surveillance.

In 2013, Murayama et al. reported the antiseptic susceptibility of canine-derived *S. pseudintermedius* isolates in Japan (8); however, no follow-up studies have been conducted to date. Therefore, in this study, we performed antiseptic susceptibility testing and polymerase chain reaction (PCR) screening for antiseptic resistance-associated genes in *S. pseudintermedius* and *S. coagulans* isolates obtained from canine pyoderma cases in Japan in 2023. Among these isolates, we recorded low minimum inhibitory concentrations (MICs) for chlorhexidine gluconate, chlorhexidine acetate, and benzalkonium chloride, although a certain degree of inter-strain variation was observed. We identified one *S. coagulans* isolate harboring a plasmid carrying the *smr* (*qacC*) gene, which represents only the second report of *smr* in this species worldwide and the first in Japan. In light of these findings, we performed an in-depth genomic analysis of this isolate, including hybrid genome sequencing.

To the best of our knowledge, this study is the first to investigate the phylogenetic structure of *S. coagulans* using public genome data and to perform hybrid genome sequencing of an *smr*-positive strain (SC18), for which we report the complete genome and demonstrate that the *smr* gene is plasmid-borne in *S. coagulans*.

## 2 Materials and methods

### 2.1 Bacterial isolates

In total, 100 clinical isolates, including *S. pseudintermedius* (*n* = 60) and *S. coagulans* (*n* = 40), were obtained from canine superficial pyoderma cases at different veterinary clinics in Japan between June and July 2023 (Supplementary Table 1). The isolates were cultured on the Pearlcore Mannitol Salt Agar (Eiken Chemical Co., Ltd., Tokyo, Japan) at a companion animal-focused bacteriology testing facility (1s Co., Ltd., Kanagawa, Japan). Species-level identification was performed using a MALDI Biotyper (Bruker Daltonics, Billerica, MA, USA) and multiplex PCR targeting *the nuc gene* (9). As an alternative to oxacillin susceptibility testing, given that *mecA* is considered a more reliable marker of methicillin resistance in *S. pseudintermedius* and *S. coagulans*, we detected this gene via PCR, as previously described (10).

### 2.2 Antiseptic susceptibility testing

Antiseptic susceptibility testing was performed as described previously (11). Briefly, 20% (w/v) chlorhexidine digluconate solution (Sigma-Aldrich, Saint Louis, MO, USA) and solid chlorhexidine diacetate (Tokyo Chemical Industry Co., Ltd., Tokyo, Japan) were used to prepare two-fold serial dilutions of 0.125–64 μg/mL. A 10% (w/v) aqueous solution of benzalkonium chloride (Alinamin Pharmaceutical Co., Ltd., Tokyo, Japan) was used to prepare dilutions from 0.5 to 12 μg/mL in 0.5 μg/mL increments. This range was selected based on the findings of a previous study (8), in which MIC values between 1 and 4 μg/mL were recorded for most of the assessed isolates. MICs were determined using the broth microdilution method, in accordance with the Clinical and Laboratory Standards Institute guidelines (M07-A11, 11th Edition) (12). Following previously described classification criteria (11), isolates with MIC values ≤ 4.0 μg/mL were categorized as having low MICs, whereas those with higher recorded MICs were categorized as having high MICs.

### 2.3 Antiseptic resistance-associated genes detection via PCR

PCR detection of antiseptic resistance-associated genes (*qacA/B*, *smr*) was performed as previously described (8, 13, 14). Gene-specific primers were used for amplification, and their sequences are listed in Supplementary Table 2. Template DNA was prepared as previously described (9) by suspending a single colony in TE buffer containing achromopeptidase (FUJIFILM Wako Pure Chemical Corporation, Osaka, Japan) and incubating at 55°C for 10 min.

### 2.4 DNA extraction and whole-genome sequencing

Whole-genome sequencing of *S. coagulans* SC18 was conducted by Genome-Lead Co., Ltd. (Kagawa, Japan) using a hybrid approach combining Illumina short-read and Nanopore long-read sequencing. DNA was extracted from bacterial cells via enzymatic lysis, as previously described (15). For Nanopore sequencing, genomic DNA (1,000 ng) was processed using a short-read liminator XS (Circulomics, Baltimore, MD, USA), followed by library preparation using a Ligation Sequencing Kit (SQK-LSK110; Oxford Nanopore Technologies, Oxford, UK) and sequencing using the MinION flow cell (FLO-MIN106 R9.41 revD) with the GridION X5 platform (Oxford Nanopore Technologies). Base calling was performed using MinKNOW (v.23.07.5) and Guppy (v.7.0.9) (16), according to the manufacturer’s instructions. Illumina sequencing libraries were prepared using the Illumina DNA Prep (M) Tagmentation Kit (formerly Nextera DNA Flex; Illumina, San Diego, CA, USA), according to the manufacturer’s protocol. Paired-end sequencing (151 × 2 cycles) was performed using the NovaSeq 6,000 instrument (Illumina). Finally, base calling, demultiplexing, and adapter trimming were performed using BCL Convert (v3.9; Illumina).

### 2.5 Genome assembly and bioinformatics analysis

Sequencing data were quality filtered by removing the low-quality short reads using standard thresholds. Hybrid assembly of the filtered short and long reads was performed using Unicycler (17). Genome completeness and contamination were evaluated using BlobTools (18), and taxonomic classification was confirmed using the Genome Taxonomy Database (19). The genome showed 97.8% average nucleotide identity with *S. coagulans* strain 1,031,336 based on the Nucleotide-Basic Local Alignment Search Tool (BLASTn) analysis. Genome polishing was conducted using Pilon (20) (short reads) and Racon (21) (long reads), followed by structural validation using BBMap (22).

### 2.6 Identification of plasmids

Assembled plasmid sequences were analyzed using BLASTn searches against the NCBI nucleotide database to identify similarities with previously reported plasmids. Plasmid identification and assessment of phylogenetic relatedness were based on sequence identity and coverage values obtained from the BLAST results.

### 2.7 Phylogenetic and clustering analyses

Next, a core genome alignment-based phylogenetic tree of *S. coagulans* was constructed using publicly available genome data with the kSNP3.0 algorithm (23), without a reference genome. Genetic population structure analysis was performed via Bayesian hierarchical clustering with the FastBAPS package (v.1.0.8) (24) in R v4.4.2 environment (25) to classify the isolates into genetically similar clusters. The number of clusters (K) was inferred automatically using optimise.baps prior, which selects the most appropriate population structure based on the input SNP alignment. Additionally, a maximum likelihood (ML) phylogenetic tree was constructed using RAxML-NG v.1.0.1 (26), with the best-fit model inferred using ModelTest-NG v.0.1.7 (27) and 100 bootstrap replicates. Subsequently, the ML tree was midpoint-rooted and visualized using FigTree v.1.4.4 (28).

### 2.8 In silico resistance gene detection

Antimicrobial resistance genes were identified using Abricate (v.1.0.1) (29) and ResFinder (v.4.6.0) (30). Quinolone resistance-determining region mutations were detected using PointFinder (v.4.1.11) (31). Methicillin-resistance gene *mecA* (NG_047936.1) and antiseptic resistance-associated genes *qacA* (AB566411.1), *qacB* (AF535087.1), and *smr* (NC_005024.1) were identified via in silico analysis using the Nucleotide-Basic Local Alignment Search Tool against the assembled genome sequences.

### 2.9 Circular genome visualization and comparative plasmid analysis

CGView (v.1.0) (32) was used to visualize the genome structure of *S. coagulans* SC18, and genome annotation was performed using Prokka (v.1.14.6) (33). In addition to the chromosome, we obtained two small circular contigs carrying replication initiation protein (rep) genes with a GC content of <30%, which is lower than that of staphylococcal chromosomes (30–40%), indicating that these sequences might be plasmids. BLASTn analysis revealed a high similarity to known plasmids, confirming their identification as plasmids. Comparative analysis of *smr*-positive plasmids was performed using Easyfig v2.2.5 (34), and homologous regions between the plasmids were visualized using gradient gray shading. For comparative analysis, we used pST827 (Z37964.1) and pKSE124-2-5 (AP028327.1), which were the top BLASTn hits identified in the search described in Section 2.6 and represented type III short rolling-circle (RC) replicating plasmids, similar to the plasmid carried by SC18.

### 2.10 Antimicrobial susceptibility testing

Inhibition zone diameters of amoxicillin/clavulanic acid, cephalexin, cefpodoxime proxetil, gentamicin, erythromycin, clindamycin, doxycycline, minocycline, enrofloxacin, chloramphenicol, and sulfamethoxazole/trimethoprim were determined using the KB/VKB discs (Eiken Chemical Co., Ltd.), according to the manufacturer’s instructions, via disc diffusion. Cefovecin disks were provided by Zoetis Japan Co., Ltd. (Tokyo, Japan). Antimicrobial susceptibility was assessed according to the Clinical and Laboratory Standards Institute guidelines (32^nd^ Edition: M100 (35) and 4th Edition: VET08 (36)) and EUCAST v.12.047 (37). In cases where interpretive criteria were not available, we followed the manufacturer’s instructions (Eiken Chemical Co., Ltd.). The interpretive criteria applied to each antimicrobial agent were as follows: amoxicillin/clavulanic acid, Eiken Chemical Co., Ltd.; cephalexin, Eiken Chemical Co., Ltd.; cefpodoxime proxetil, CLSI VET08, 4th ed.; cefovecin, CLSI VET08, 4th ed.; gentamicin, Eiken Chemical Co., Ltd.; erythromycin, CLSI M100, 32nd ed.; clindamycin, CLSI M100, 32nd ed.; doxycycline, CLSI M100, 32nd ed.; minocycline, EUCAST v12.0; enrofloxacin, CLSI VET08, 4th ed.; chloramphenicol, CLSI M100, 32nd ed.; sulfamethoxazole/trimethoprim, CLSI M100, 32nd ed.

### 2.11 Statistical analysis

To assess the association between the presence of antiseptic resistance-associated genes and *mecA*, we performed an analysis using Fisher’s exact test in R v4.4.3. A *p*-value of less than 0.05 was considered to be indicative of statistical significance.

## 3 Results

### 3.1 Antiseptic susceptibility and resistance genes in canine-derived *Staphylococcus* isolates

In 2023, we collected 60 *S. pseudintermedius* and 40 *S. coagulans* strains from canine superficial pyoderma cases in Japan (Supplementary Table 1). Among these, 33 *S. pseudintermedius* and 27 *S. coagulans* isolates were *mecA*-positive (Supplementary Table 3). To determine their susceptibility to antiseptic chlorhexidine, MICs were measured using the broth microdilution method (Table 1 and Supplementary Table 4). MIC_90_ was 1.0 μg/mL for chlorhexidine gluconate and 0.5 μg/mL for chlorhexidine acetate. Importantly, all isolates exhibited low MICs (≤ 2.0 μg/mL) to chlorhexidine, regardless of species or *mecA* status.

**Table 1:** Minimal inhibitory concentrations (MICs) of chlorhexidine compounds for methicillin-susceptible *Staphylococcus pseudintermedius* (MSSP), methicillin-resistant *S. pseudintermedius* (MRSP), methicillin-susceptible *Staphylococcus coagulans* (MSSC), and methicillin-resistant *S. coagulans* (MRSC) strains.

MIC (μg/ml)	CHG				CHA			
	MSSP	MSSC	MRSP	MRSC	MSSP	MSSC	MRSP	MRSC
≦0.125	0	0	0	0	1	0	0	0
0.25	0	0	0	0	14	10	20	20
0.5	20	2	17	10	12	3	13	7
1	7	11	16	16	0	0	0	0
2	0	0	0	1	0	0	0	0

CHG, chlorhexidine gluconate; CHA, chlorhexidine acetate.

PCR screening for antiseptic resistance-associated genes revealed that one methicillin-susceptible *S. coagulans* isolate (SC18) carried *smr* (*qacC*; Supplementary Table 3). This gene primarily confers reduced susceptibility to quaternary ammonium compounds rather than to chlorhexidine (38). Therefore, susceptibility testing was performed for benzalkonium chloride, for which we recorded a notably low MIC of ≤0.5 μg/mL against SC18 (Supplementary Table 4). The isolates in this study showed an MIC₉₀ of 1.0 μg/mL (Table 2).

**Table 2:** Minimal inhibitory concentrations (MICs) of benzalkonium chloride against methicillin-susceptible *Staphylococcus pseudintermedius* (MSSP), methicillin-resistant *S. pseudintermedius* (MRSP), methicillin-susceptible *Staphylococcus coagulans* (MSSC), and methicillin-resistant *S. coagulans* (MRSC) strains.

MIC (μg/ml)	BKC			
	MSSP	MSSC	MRSP	MRSC
≦0.5	17	23	16	23
1	10	3	11	3
1.5	0	1	3	1
2	0	0	1	0
2.5	0	0	1	0

Susceptibility data for BKC was not determined for one MRSP isolate. BKC, benzalkonium chloride.

### 3.2 Phylogenetic analysis of *S. coagulans* and global distribution of antiseptic resistance-associated genes

**Figure 1:**
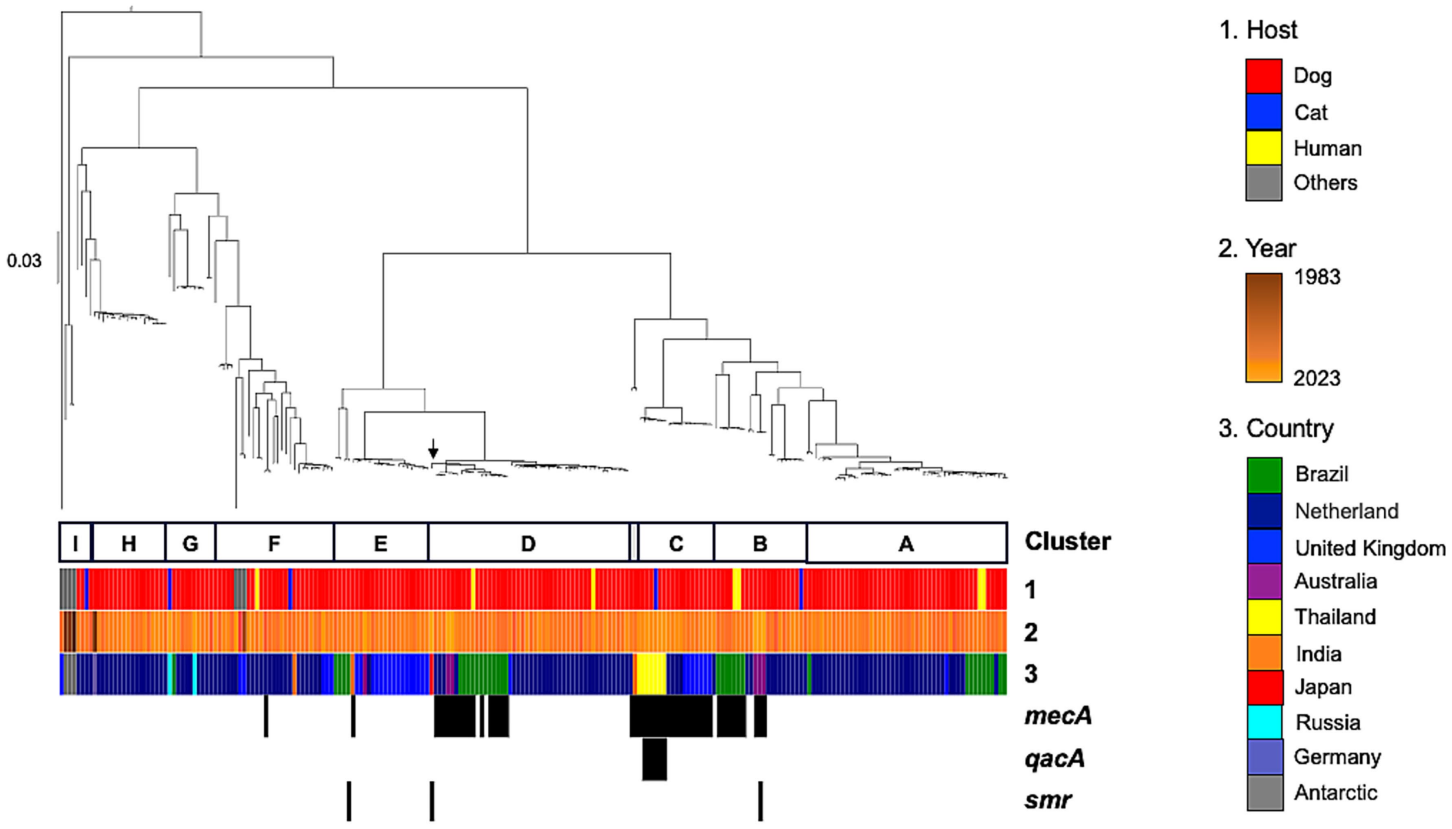
Population structures of SC18 and 227 publicly available *Staphylococcus coagulans* isolates. A parsimony tree was constructed using kSNP3 based on 18,591,633 single nucleotide polymorphism (SNP) sites identified from the pangenome of 228 isolates. Branch lengths represent the number of SNP differences. Sequence clusters (A–I) were identified via Bayesian analysis of population structure (BAPS) using FastBAPS. Heatmap columns represent the following: Column 1 = host species, column 2 = isolation year, and column 3 = isolation country. Black boxes indicate the presence of antimicrobial or antiseptic resistance-associated genes.

To determine the phylogenetic position of SC18, we performed whole-genome sequencing and constructed a phylogenetic tree using the kSNP algorithm incorporating 227 publicly available *S. coagulans* genomes retrieved from the National Center for Biotechnology Information database (Figure 1). Bayesian population structure analysis was performed using the fastbaps v1.0.8 package in R, based on the core genome SNPs. The optimal number of clusters (K) was inferred automatically using optimise.baps prior, which selects the most appropriate population structure from the data. This analysis grouped all isolates into nine major clusters (A–I). SC18 was classified into cluster D, the largest cluster comprised 48 isolates (Supplementary Table 5).

To analyze the resistance (−associated) genes distribution, local Basic Local Alignment Search Tool searches were performed against all genomes using the reference sequences for *mecA* (NG_047936.1), *qacA* (AB566411.1), *qacB* (AF535087.1), and *smr* (NC_005024.1). The results revealed that 21.1% (*n* = 48) of the genomes were *mecA*-positive, whereas 3.9% (*n* = 9) carried antiseptic resistance-associated genes (Figure 1 and Supplementary Table 5). Specifically, *qacA* was detected in 2.6% (*n* = 6) and *smr* in 1.3% (*n* = 3) of the isolates.

Although *mecA* was predominantly found in phylogenetic clusters B–D, the distribution of antiseptic resistance-associated genes was characterized by a geographical trend, with *qacA* detected exclusively in isolates from Thailand and *smr* identified in isolates from Japan, Australia, and Brazil.

Among the nine isolates carrying antiseptic resistance-associated genes (*qacA* or *smr*), seven (77.8%) also harbored *mecA*. Statistical analysis using Fisher’s exact test revealed a significant association between the presence of antiseptic resistance-associated genes and *mecA* (*p* = 0.00042, odds ratio = 14.18). All six *qacA*-positive isolates were *mecA*-positive (*p* = 0.000067, odds ratio = ∞), indicating a strong association. In contrast, no significant association was observed between *smr* and *mecA* (*p* = 0.51). These findings provide statistical support for the co-occurrence of antiseptic and antibiotic resistance genes, particularly *qacA*, although the sample size was limited.

### 3.3 Complete genome analysis of the *smr*-positive *S. coagulans* isolate SC18

**Figure 2:**
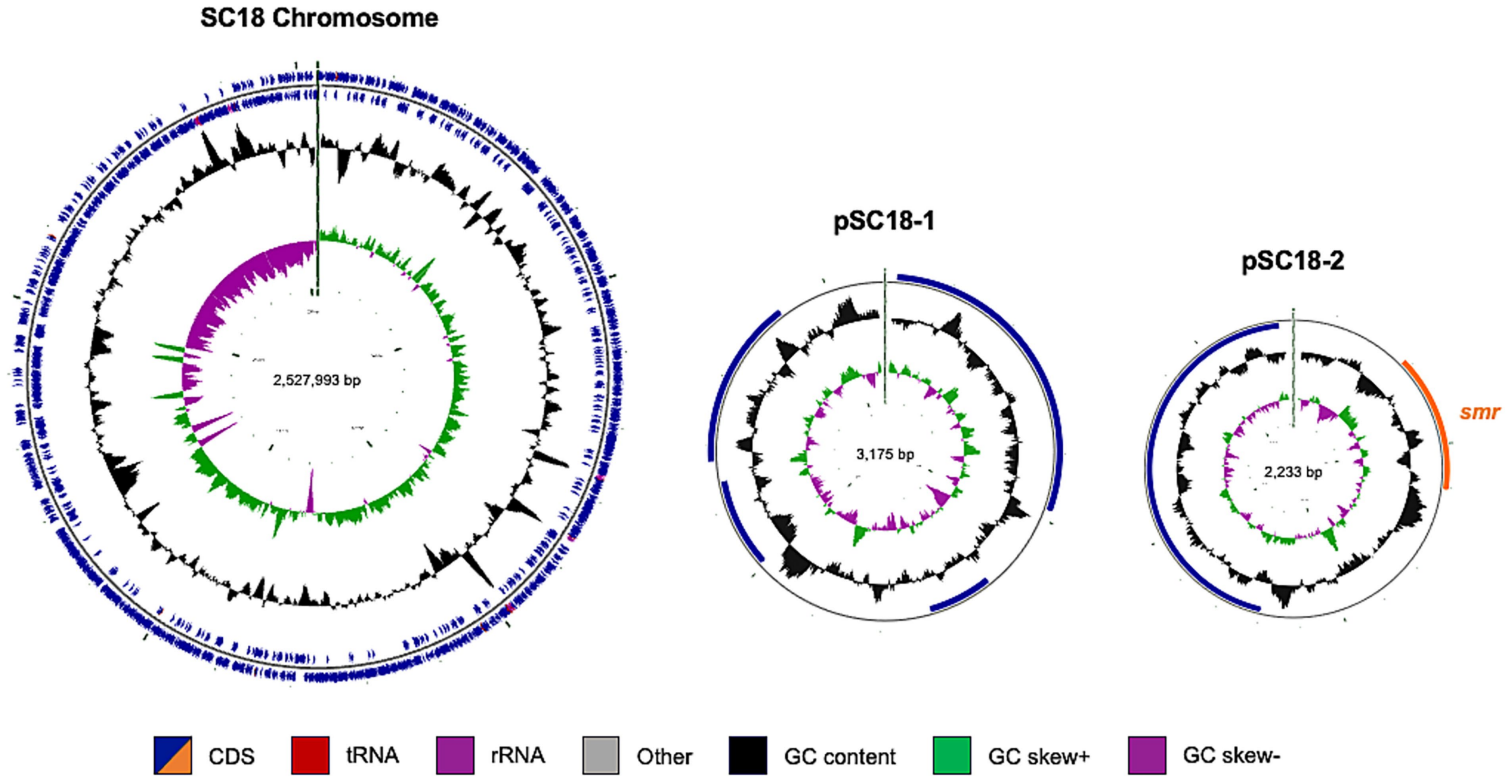
Genome structure of the *smr*-positive *S. coagulans* isolate SC18. The complete genome of SC18 comprised a circular chromosome (2,527,993 bp) and two plasmids, pSC18-1 (3,175 bp) and pSC18-2 (2,233 bp). Circular genome maps were generated using CGView (v1.0), visualizing the coding sequences, structural RNAs, GC content, and GC skew. Functional elements are color-coded, as indicated in the legend.

To further characterize SC18, we performed hybrid sequencing to determine the complete genome of *S. coagulans* SC18. This strain possessed a single circular chromosome and two plasmids, pSC18-1 and pSC18-2, with *smr* located in pSC18-2 (Figure 2).

pSC18-1 was 3,175 bp in length and encoded a single *rep* gene and three additional genes predicted to encode hypothetical proteins with unknown functions. BLASTn analysis revealed that pSC18-1 had 94.36% sequence identity and 63% coverage with an unnamed plasmid (CP094737.1) from *S. delphini* strain IVB6222, which was isolated from a camel in Kenya (39). However, no antimicrobial resistance-or virulence-associated genes were identified in this plasmid.

pSC18-2 is a small plasmid of 2,233 bp containing only *smr* and *rep.* This is a type III short RC replicating plasmid previously characterized in staphylococci (40).

**Figure 3:**
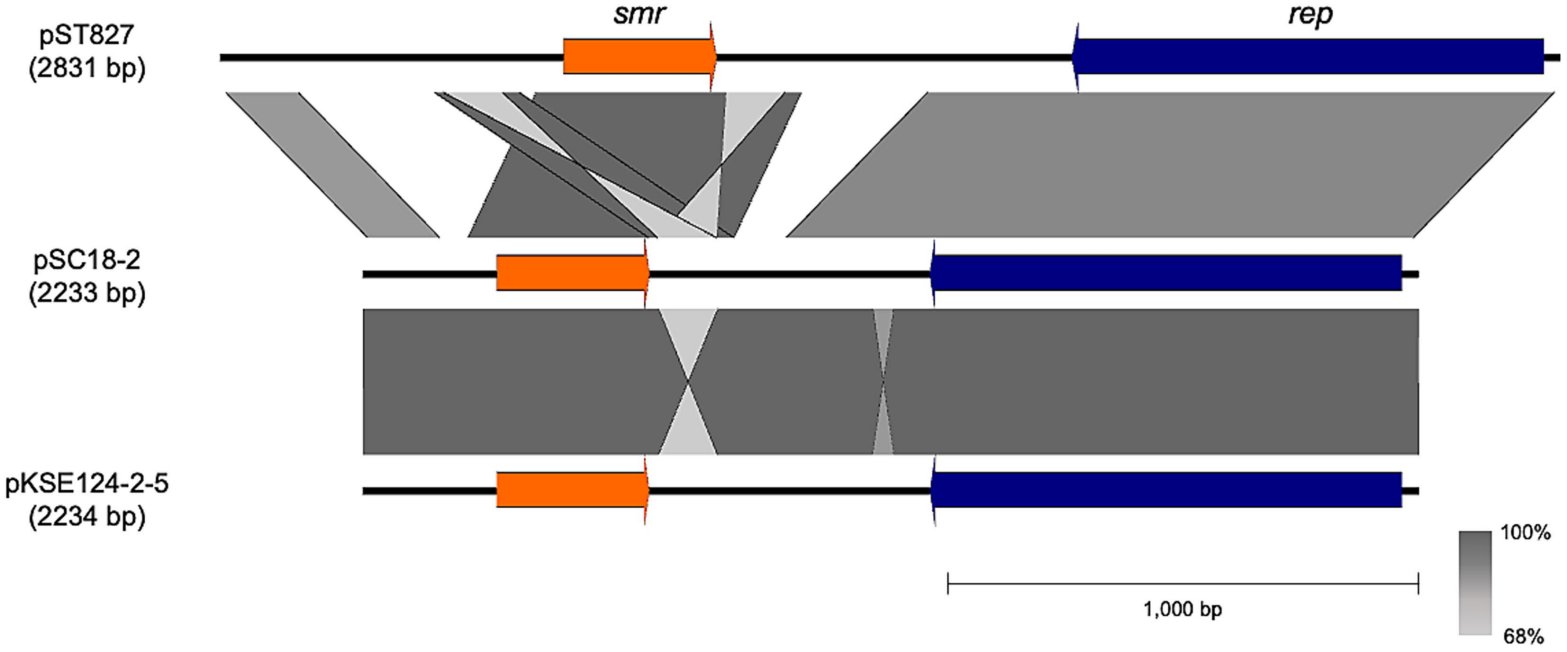
Comparative analysis of *smr*-positive type III short rolling-circle replicating plasmids. The figure was generated using Easyfig v2.2.5. Plasmid pSC18-2 from SC18 was compared with two known *smr*-positive plasmids, pST827 (Z37964.1) and pKSE124-2-5 (AP028327.1). Open reading frames (ORFs) are shown as arrows, with *smr* in orange and *rep* in navy blue. Sequence homology between plasmids is illustrated with gradient shades of gray, representing 68–100% nucleotide identity. The scale bar indicates 1,000 bp.

Comparative alignment revealed that pSC18-2 shared 70.5% sequence identity with pST827 (Z37964.1), a previously described type III short RC replicating plasmid from *Staphylococcus* spp. (41). Moreover, pSC18-2 exhibited high sequence similarity (99.7%) to pKSE124-2-5 (AP028327.1), a plasmid isolated from the *S. epidermidis* KSE124-2 strain obtained from a human in Hiroshima (Japan) in 2016 (Figure 3). Given the high similarity, pSC18-2 is considered genetically identical to pKSE124-2-5; however, we refer to this plasmid as pSC18-2 to enable a clear distinction for comparative analyses.

SC18 did not harbor any additional antimicrobial resistance genes and remained susceptible to clavulanic acid/amoxicillin, cephalexin, cefpodoxime proxetil, cefovecin, gentamicin, erythromycin, clindamycin, doxycycline, minocycline, chloramphenicol, and sulfamethoxazole/trimethoprim (Supplementary Tables 6, 7). However, this strain harbored several mutations in chromosomal quinolone resistance-determining regions, including GyrA S84F, ParC S80I and E84D, and ParE D432E and P535Y, conferring it resistance to enrofloxacin. This was the only antimicrobial-resistant phenotype observed in SC18.

## 4 Discussion

In this study, we assessed the antiseptic susceptibility of companion animal isolates in Japan, which has not been comprehensively studied in recent years. Among the isolates assessed, we recorded low MICs to chlorhexidine compounds and benzalkonium chloride, similar to a previous report (8). Additionally, we identified an *smr*-positive *S. coagulans* strain (SC18) for the first time in Japan. To the best of our knowledge, this is the second report of an *smr*-positive *S. coagulans* strain worldwide. Whole-genome analysis revealed that SC18 is a member of the major phylogenetic lineage of *S. coagulans* and harbors a short RC-replicating plasmid (pSC18-2) carrying the *smr* gene. This is the first report of a plasmid-borne *smr* gene in *S. coagulans*.

The *smr* gene primarily confers reduced susceptibility to quaternary ammonium compounds rather than to chlorhexidine (38). However, for SC18, we obtained low MIC values for both chlorhexidine and benzalkonium chloride, consistent with a previous report in Australia (42). Therefore, it is plausible that presence of *smr* does not confer a detectable reduced phenotypic susceptibility to *S. coagulans*. However, reduced susceptibility can emerge depending on antiseptic usage (43), which could be attributed to an increase in gene copy number or other regulatory changes (43, 44), highlighting the importance of the continuous surveillance of antiseptic susceptibility and resistance-associated gene carriers.

Short *smr*-carrying RC replicating plasmids have been increasingly detected in recent years, particularly in Asia (45), raising concerns regarding their uncontrolled dissemination. In Japan, the isolation rate of *smr*-positive plasmids has increased from 3.4% in the late 1990s to 10.8% in 2003 (45, 46). The plasmid pSC18-2 identified in this study exhibits 99.7% sequence identity with pKSE124-2-5, which was isolated in 2016 from a human-derived *S. epidermidis* strain in Hiroshima, Japan, and is accordingly considered genetically identical. This finding provides evidence of the transmission of plasmids from humans to companion animals. Moreover, given that pKSE124-2-5 originated from Hiroshima and pSC18-2 originated from Osaka, it is likely that this plasmid had already been disseminated across western Japan or even more broadly within the country. Short RC replicating plasmids exhibit high mobility, and further dissemination is anticipated.

In this study, we mainly aimed to determine the chlorhexidine susceptibility status of canine pyoderma-derived isolates. We used the PCR screening methodology of Murayama et al. (8), focusing on *qacA/B* and *smr*. To date, many other antiseptic resistance-associated genes, such as *qacE*, *qacG*, *qacH*, and *qacJ*, which are mainly associated with reduced susceptibility to quaternary ammonium compounds, have not been comprehensively evaluated. Therefore, future studies should incorporate more inclusive methods to detect these genes. Furthermore, investigation of *smr* levels and relationship between antiseptic exposure and reduced phenotypic susceptibility is necessary to enhance our understanding of the mechanisms underlying reduced susceptibility at the phenotypic level.

## 5 Conclusion

In conclusion, this study provided updated data on the antiseptic susceptibility of companion animal-derived staphylococci in Japan, revealing that the identified isolates generally susceptible to chlorhexidine and benzalkonium chloride, for which we recorded low MIC values. Furthermore, to the best of our knowledge, this study is the first to isolate and identify an *smr*-positive *S. coagulans* strain (SC18) harboring the short RC plasmid, pSC18-2. Although SC18 showed no reduced phenotypic susceptibility, the presence of highly mobile *smr-*carrying plasmids suggests its potential risk of dissemination. Overall, our findings underscore the need for continuous surveillance and broader genetic and functional investigations of antiseptic reduced susceptibility in companion animals.

## Data Availability

The sequencing reads of *S. coagulans* SC18 generated in this study have been deposited in GenBank/EMBL/DDBJ under the run accession numbers DRR660260–DRR660261.
